# Posterior Chamber Hemorrhage during Fluorescein Angiography

**DOI:** 10.1155/2015/728070

**Published:** 2015-06-28

**Authors:** Manuel A. P. Vilela

**Affiliations:** ^1^Department of Specialized Medicine, Ophthalmology, Federal University of Pelotas, Avenida Duque de Caxias 250, Fragata, 96001970 Pelotas, RS, Brazil; ^2^Post-Graduation Program in Health Sciences: Cardiology, Instituto de Cardiologia/Fundação Universitária de Cardiologia, Porto Alegre, RS, Brazil

## Abstract

This paper provides the first reported case of acute posterior chamber hemorrhage during fluorescein angiography (FA). This is a case review with serial color photographs of the anterior segment. A 76-year-old male was referred for angiographic control of age-related macular degeneration. He was pseudophakic OU, BCVA 20/40 OU. He had mild hypertension, but not diabetes. He had had two previous angiograms without adverse effects. Difficulty was experienced in obtaining the images owing to a progressive reduction in the transparency of the media. A dense hemorrhage in the posterior chamber of the right eye was found, involving the visual axis. Thorough biomicroscopy, gonioscopy, and ultrasonic biomicroscopy showed that part of one of the haptics of the right intraocular lens (IOL) was touching and tearing the posterior face of the iris, without any visible synechiae, iris, or angle neovascularization. Anterior segment FA and posterior ultrasonography were normal. No similar case has been described in the literature involving dense progressive bleeding located in the capsular bag and posterior chamber, without any detectable triggering ocular event other than mydriasis and fluorescein injection. Contact of the iris or sulcus with part of the intraocular lens, aggravated by the intense use of mydriatics during the FA procedure, probably caused bleeding to happen.

## 1. Introduction 

Fluorescein angiography (FA) is a very safe procedure, with infrequent complications and low morbidity [1–4]. Besides the expected effects of fluorescein injection (e.g., impregnation of the skin and urine, sneezing, and metallic taste), other reactions that require immediate treatment may occur. The most common ones are mild nausea (0.8%–15%) and vomiting (1%–4%); moderate reactions include urticaria (1.2%) and syncope (0.29%); severe reactions like anaphylactic shock, hypotension, respiratory failure, pulmonary edema, seizures, cardiovascular collapse, and arrhythmias are reported in 0.02 to 0.007% of cases. Death due to the procedure is estimated to happen in 0.00045% of cases (1 : 49557–221781). There are no reports in the literature of severe ocular adverse effects related to this procedure. We report a case of atypical posterior chamber hemorrhage immediately following FA.

## 2. Case Report 

**Figure 1:**
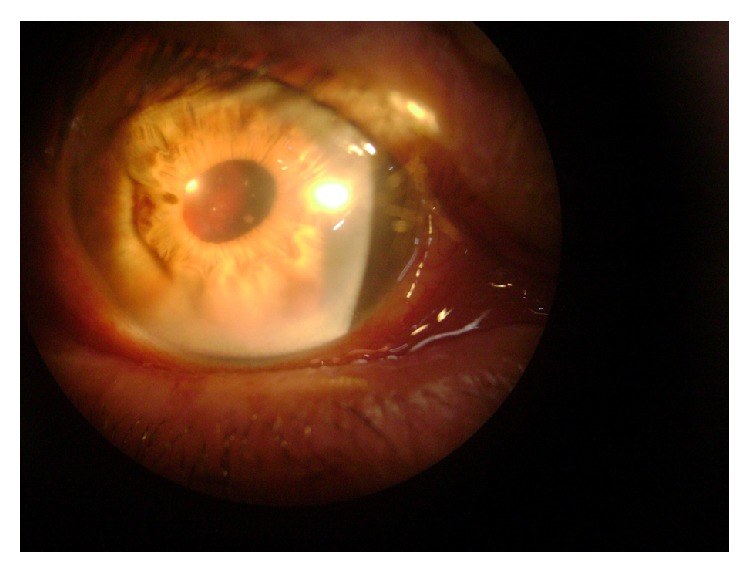
Presence of acute bleeding in the posterior chamber involving the visual axis.

**Figure 2:**
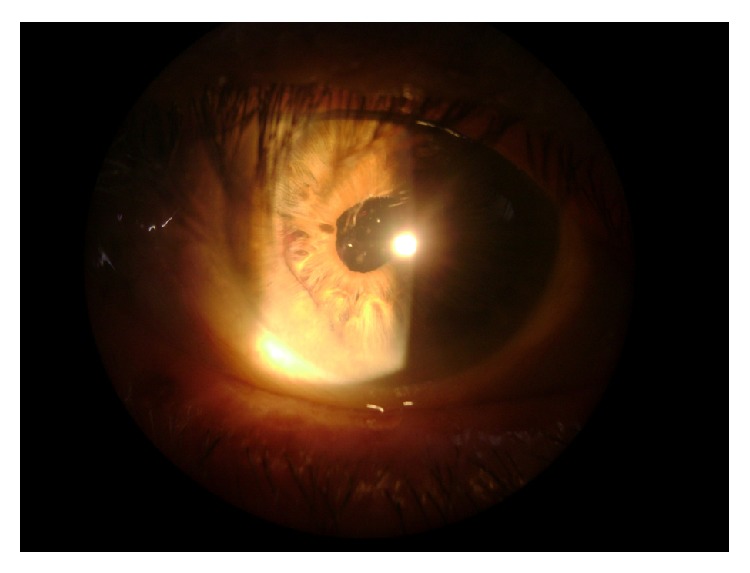
After 24 hours complete reabsorbtion of blood with total visual recovery.

A 76-year-old male was referred for angiographic control of age-related macular degeneration. He was pseudophakic in both eyes (OU) (since 2007—phacoemulsification with foldable implant, “clear cornea” technique), having best corrected visual acuity (BCVA) 20/40 OU and intraocular pressure (IOP) 11 mmHg OU. He had mild hypertension. He had had two previous angiograms without complications. In November 2009 he underwent FA at our clinic, following pupil dilation using tropicamide 1% plus 2.5% phenylephrine solution (3 instillations 30 minutes before the procedure). Before the photos were taken, he complained of floaters in the right eye (RE). Difficulty was experienced in obtaining the images owing to a progressive reduction in the transparency of the media. The images obtained during the procedure showed great confluence of drusen in the posterior pole of the right eye, with no signs of papillary, retinal, or choroidal neovascularization. Owing to the worsening of symptoms he was reexamined before leaving the clinic. A dense hemorrhage in the posterior chamber of the right eye was found, enveloping the visual axis (Figure 1). Bed rest with raised head, bilateral eye occlusion, and topical corticosteroids were prescribed. He was seen 48 hours later, when eye examination showed reabsorption of most of the blood, thus releasing the optical axis (Figure 2). His visual acuity had returned to prior values. He was submitted to thorough biomicroscopy, gonioscopy, and ultrasonic biomicroscopy (UBM), which showed that part of one of the haptics of the right intraocular lens (IOL) was touching and tearing the iris, without any visible synechiae, iris, ciliary body lesion, or angle neovascularization. The corneal incision had no abnormal vessels either. It was impossible to rule out any adhesive focus between the lens capsular bag and the posterior iris. Anterior segment FA and posterior ultrasonography were normal.

## 3. Discussion

We found no similar case in the literature involving dense progressive bleeding located in the capsular bag and posterior chamber, without any detectable triggering ocular event other than mydriasis and fluorescein injection. Intraocular hemorrhage months or years after cataract surgery has been described as SWAN syndrome, in which stromal vascular growth is detectable and produces recurrent bleeding approximately 36–48 months after surgery [5]. However, this does not seem to be the case here. Another possibility is that it may have originated in the ciliary body [6, 7], but gonioscopy and UBM were entirely normal. We suppose that the ocular hemorrhage this patient presented was due to pharmacologic mydriasis and not to fluorescein, but its cause remains uncertain, considering that the patient had had his pupils dilated many times before with no adverse effects. Contact of the posterior face of the iris or sulcus with a portion of the intraocular lens, aggravated by the intense use of mydriatics during the FA procedure, probably caused bleeding rather than this being related to fluorescein injection.
